# Supplementary figures and images for: An evaluation protocol of ‘Replicability Model’ project for detection and treatment of leprosy and related disability in Chhattisgarh, India
Source: PLoS One. 2023 Oct 18;18(10):e0275763. doi: 10.1371/journal.pone.0275763 (PMC10584107; doi:10.1371/journal.pone.0275763)

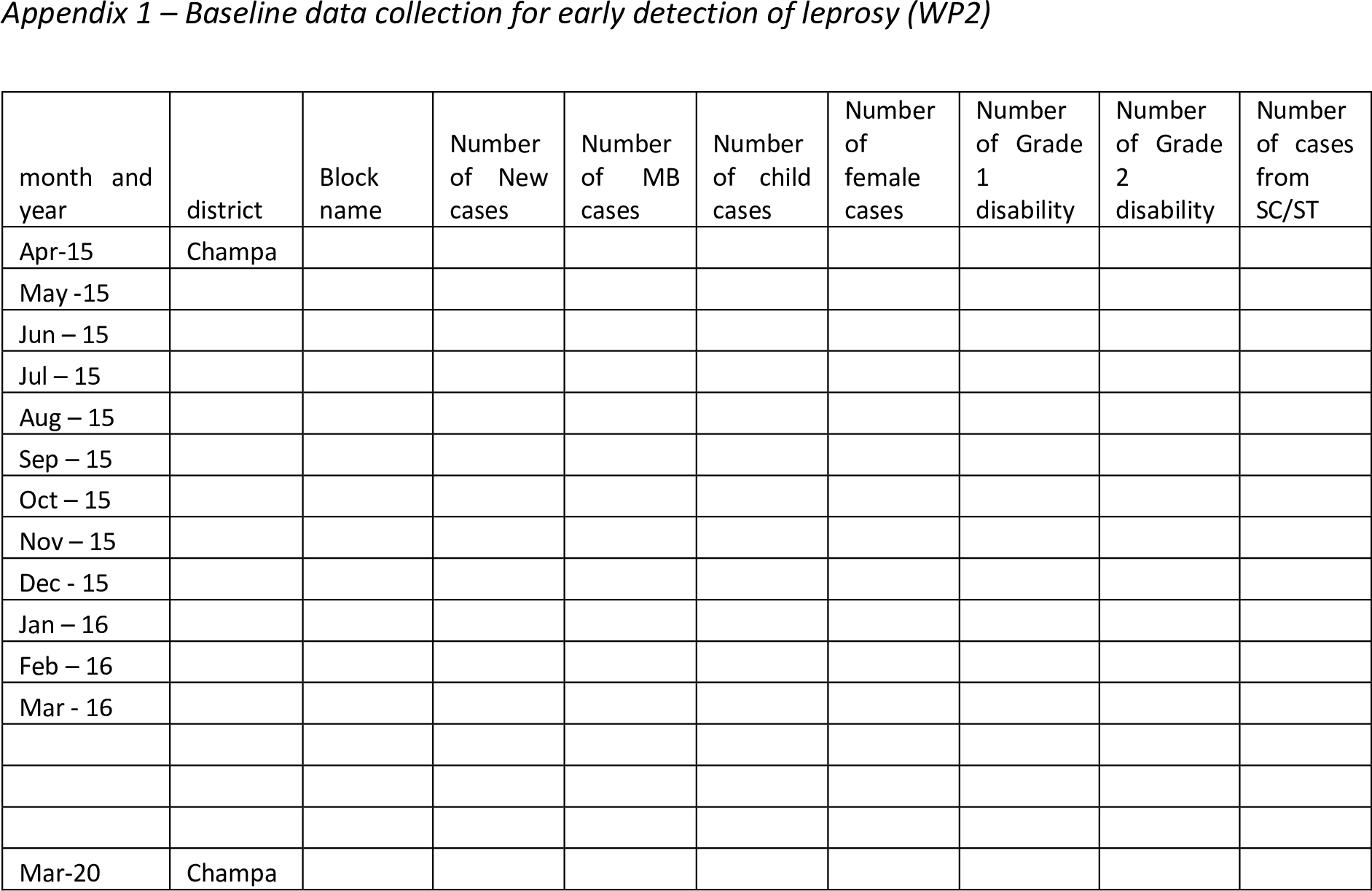

Supplement: S1 Appendix — (TIF) [file pone.0275763.s001.tif]

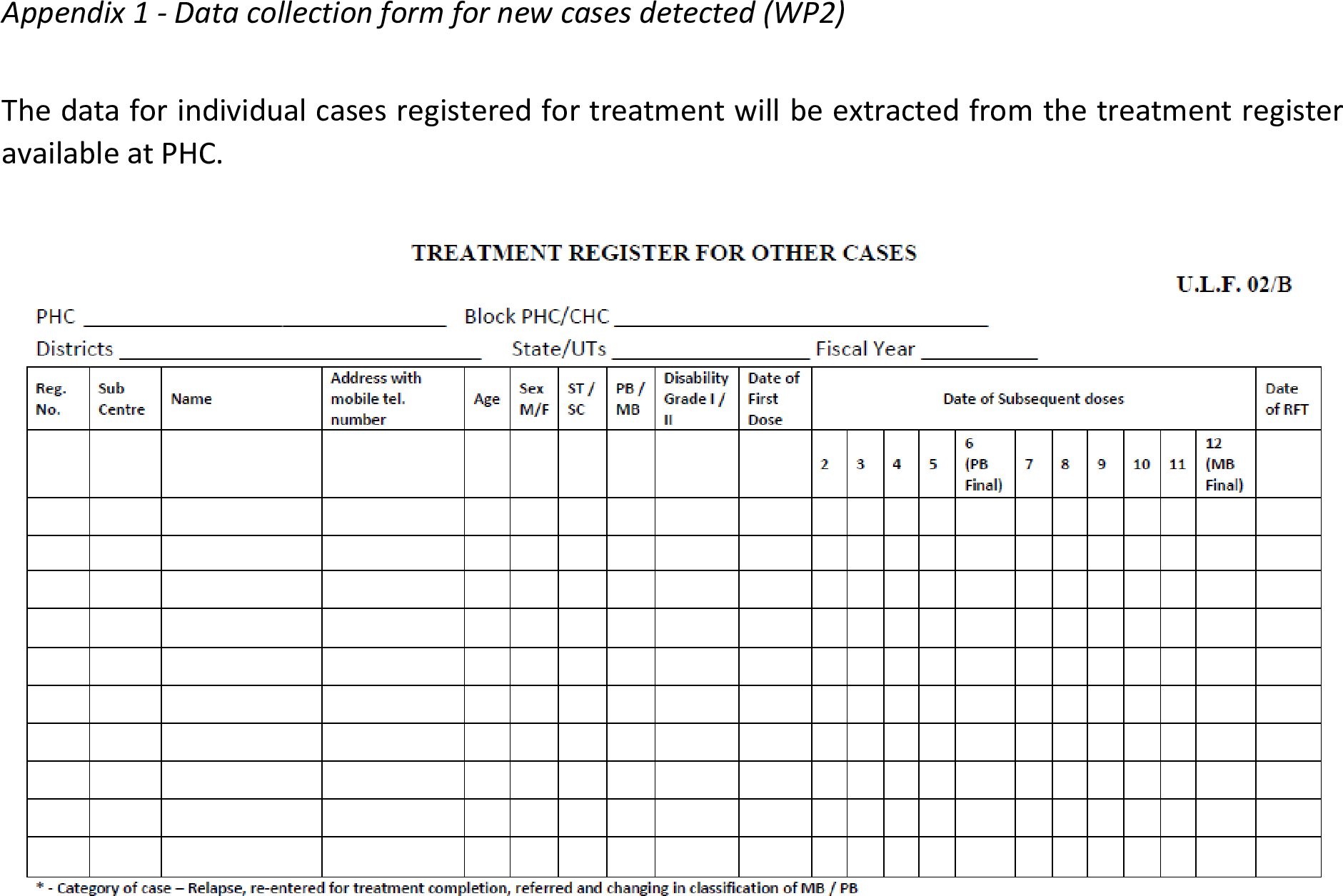

Supplement: S2 Appendix — (TIF) [file pone.0275763.s002.tif]
