# Supplementary material for: An evaluation protocol of ‘Replicability Model’ project for detection and treatment of leprosy and related disability in Chhattisgarh, India
Source: PLoS One. 2023 Oct 18;18(10):e0275763. doi: 10.1371/journal.pone.0275763 (PMC10584107; doi:10.1371/journal.pone.0275763)
Supplement: S1 File — (DOCX) [file pone.0275763.s003.docx]

**Supplementary file 1: TIDieR description of replicable model project interventions**

1. Name of the intervention: Replicable model project
2. Why (describe the logic or rationale of the intervention):

The overall aim of the replicable model project is to reduce the disability rate among newly diagnosed leprosy patients in Janjgir Champa district. This will be achieved through various interventions, for example, raising awareness about leprosy in the community, and health promotion and building capacity of health workers to enable early detection and management of leprosy and its complications. The aim is for these interventions to be replicated elsewhere. At present, there are no evidence-based models, which can be replicated in other settings. Interventions that are evidence-based and implemented through the national health program for leprosy are more likely to be sustainable and can be replicated elsewhere than stand-alone interventions.

1. What materials (describe materials used in the intervention):

There are three key interventions planned under the RM project. 1) raise awareness about leprosy in the community through a mass awareness campaign to improve early detection of leprosy, 2) develop the capacity of various levels of health care workers involved in leprosy in the district by providing training on how to suspect, diagnose and manage leprosy and its complications, and 3) improve access to rights and entitlements, reduce stigma and discrimination to promote inclusion of people affected by leprosy. The standard government-approved curriculums will be used for training different cadres of leprosy workers.

To implement these interventions:

1) We will develop new or modify existing messages on behavioural change communication (messages) to improve awareness in the community on the signs and symptoms of leprosy and the availability of leprosy-related services at various levels of healthcare facilities. The messages will be disseminated across the district using various mediums through public media campaigns which will be identified as part of the intervention in consultation with various stakeholders.

2) We will improve the capacity of health workers and volunteers across the district by:

1. Updating the existing training modules for Mitanins on leprosy addressing gaps in the content, for example, expanding information on the signs and symptoms related to lepromatous leprosy (difficult to diagnose cases).
2. Adapting the existing training curriculum on the diagnosis of leprosy and management of its complications for Medical Officers and assistant medical officers from the government and TLMTI. This adaptation will condense the training duration from the current 5-day course to a 3-day course. Education on contact tracing will be included in the training curriculum of Mitanins and Medical officers.
3. Village Health, Sanitation & Nutrition Committee (VHSNC) members will be sensitized about leprosy by Mitanins to improve awareness about leprosy in the community and they, in turn, will include leprosy in their regular health promotion activities.
4. Rashtriya Bal Swasth Karyakram (Child Health Screening and Early Intervention Services), RBSK team(18) in the community will be trained to screen and suspect signs and symptoms of leprosy among children of 18 years or less and referral to an appropriate health facility. The screening campaigns will be held across public-owned schools in the Janjgir-Champa district.
5. Develop a training curriculum for volunteers on how to suspect leprosy in the community. The volunteers are from the local community and are involved in active case detection campaigns as and when planned by the district-level authority, usually once or twice a year.
6. What and how (describe how the intervention was planned, established, and intended to be delivered):

The replicable model project staff will collate the available messages on improving awareness about leprosy and seek feedback from people who have used it on its effectiveness. In consultation with various stakeholders, the team will develop new messages or modify existing messages to improve awareness in the community. After identifying effective media in consultation with key stakeholders the implementation plan to cover the entire district will be planned along with the district-level health authority of the national leprosy program to roll out the awareness program across the district. The messages will be disseminated through public media campaigns.

The training curriculum for various cadres of healthcare workers is available. In this project, we will update the curriculum and/or adapt it where required with the help of experts available in The Leprosy Mission Trust India (TLMTI) and the Government. The training program schedule will be decided in consultation with the district-level health authority. The medical officers and assistant medical officers will be trained together, grouped according to the block where they are posted. Training of Mitanins and others will be done through master trainers and their supervisors. Master trainers regularly conduct training sessions to increase the capacity of Mitanins on various national health programs where training on leprosy will be done using updated curriculum. Mitanins in turn will train male volunteers for case detection campaign and sensitize the Village Health, Sanitation & Nutrition Committee members to include leprosy in their health promotion activities.

1. Who provided (describe the provider of the intervention):

The Replicable model project staff (RM team) will be involved in the planning, implementation, and monitoring of the awareness program. The interventions (awareness program) will be provided by and through the staff of the national leprosy eradication program (NLEP) including paramedical workers and the district nucleus team.

The training program for various cadres of health care workers will be provided jointly by master trainers, supervisors of health care workers from the district and experts from the RM team. The RM team will develop a cascade of training programs and train the trainers (master trainers). The master trainers will train all Mitanins of the district. The Mitanins will sensitize Village Health, Sanitation & Nutrition Committee members on leprosy and volunteers on how to screen for and refer suspected cases.

The RBSK (Child Health Screening and Early Intervention Services) mobile teams will be trained by RM project team, experts from The Leprosy Mission Trust India and national leprosy program on screening of children for leprosy.

Experts in leprosy from national leprosy program and The Leprosy Mission Trust India will conduct training for medical officers, assistant medical officers, and para-medical workers.

1. How (describe the modes of delivery (e.g., face to face or by some other mechanism, such as internet or telephone) of the intervention and whether it was provided individually or in a group.

See section 4.

1. Where (describe the type of location, geographical scope of the intervention):

The replicable model project intervention will be rolled out across the entire Janjgir-Champa district. See Section 2. The Janjgir-Champa District, is situated in the centre of Chhattisgarh state, surrounded by Korba in the North, Bilaspur district in the West, Baloda Bazar in the South and Raigarh district in the East. The total population of the district is 1,619,707 (according to census 2011), with a sex ratio of 986 females to one thousand males. Administratively, the urban area is divided into 4 Municipalities and 11 Nagar Panchayats. The rural area of the district is divided by 9 Community development blocks (CD blocks), which contain 631 Panchayats and 915 villages most (1) having a population between 250 to 1000. The public health system of the district consists of one district hospital, one civil hospital, 14 Community Health Centres (CHC) and 49 Primary Health Centres.

1. When and how often (describe when the intervention was provided and how long including scheduling):

The awareness program aims to cover at least 80% of villages in the district over 10-12 months. Some awareness raising initiatives like radio messages or wall paintings will continue after this time, covering the entire district.

The Mitanins (3791), VHSNC members, RBSK (Child Health Screening and Early Intervention Services) team members and volunteers involved in the active case detection campaign of the district will be trained on how to suspect leprosy and the referral process to higher health facilities for diagnosis and treatment. As mentioned above Mitanins will be trained by master trainers. A total of 40 master trainers will be trained in a 3-day program and they in turn will cascade training to all Mitanins within 6 months. The Mitanins will sensitize VHSNC members on leprosy. The medical officers (7) and assistant medical officers (65) posted in the Primary Health Centres (PHC) from the district will be trained in 8 groups based on block where they are posted. All the training programs will be conducted at the PHC or Community Health Centre (CHC) at the block level.

The medical officers and assistant medical officers will receive one off training program in 8 different groups according to the block where they are posted, and they all will be covered in 3 months.

The RBSK (Child Health Screening and Early Intervention Services) mobile teams will be covered in one off training program in the district headquarter. If required, a refresher course will be planned to depend on the feedback from trainee.

1. Planned variation (describe and provide the reason for any variation or tailoring was planned):

The media used to communicate the messages on awareness on leprosy may not be uniform across the district. For example, radio messages may reach across the district but medium such as folk dance will be used in different places where they are more effective.

The curriculum for medical and assistant medical officers will be condensed to 3-day program from the existing duration of 5 days. The training for RBSK team (Child Health Screening and Early Intervention Services) will be restricted to screening of leprosy among children.

1. Unplanned variation (describe and provide the reason for any unplanned variation or modification):

Not applicable at this stage

1. How well (describe any strategies used or actions taken to maintain fidelity of the intervention):

The RM team will be involved in planning, implementation, and monitoring of the awareness program. Standard and approved training curriculum for Mitanins, medical officers and assistant medical officers will be used. The adaptation made in the curriculum will be validated by an expert in leprosy.

The training program for master trainers, medical officers and assistant medical officers will consists of pre and post-test training test to assess their change in knowledge level.

The skill level of medical officers and assistant medical officers on skin and nerve examination will be observed and evaluated against checklist.

The skill level of para-medical worker on performing nerve function assessment will be observed and evaluated against checklist.

1. How well – delivery (describe fidelity of the intervention):

Not applicable at this stage
