# Supplementary material for: An evaluation protocol of ‘Replicability Model’ project for detection and treatment of leprosy and related disability in Chhattisgarh, India
Source: PLoS One. 2023 Oct 18;18(10):e0275763. doi: 10.1371/journal.pone.0275763 (PMC10584107; doi:10.1371/journal.pone.0275763)
Supplement: Attachment — Submitted filename: PONE-D-22-25961_reviewed.pdf [file pone.0275763.s005.pdf]

PLOS ONE

An Evaluation Protocol of ‘Replicability Model’ project for detection and treatment of leprosy and related disability in Chhattisgarh, India.

--Manuscript Draft--

Manuscript Number:	PONE-D-22-25961
Article Type:	Study Protocol
Full Title:	An Evaluation Protocol of ‘Replicability Model’ project for detection and treatment of leprosy and related disability in Chhattisgarh, India.
Short Title:	An Evaluation Protocol of ‘Replicability Model’ project for detection and treatment of leprosy.
Corresponding Author:	Joydeepa Darlong, MBBS MD<br>The leprosy mission trust India<br>New Delhi, New Delhi INDIA
Keywords:	System strengthening; early detection; Leprosy; disability; Low and middle-income countries
Abstract:	Introduction<br><br>People affected by leprosy are at increased risk of impairments and deformities from peripheral nerve damage. This mostly occurs if diagnosis and treatment is delayed and contributes to continued transmission within the community. Champa district of Chhattisgarh state in India is an endemic area with the highest national annual case detection and disability rates for leprosy. The Replicability Model is a system strengthening intervention implemented by the Leprosy Mission Trust India in Champa that aims to promote early diagnosis and treatment of leprosy, improve on-going management of the effects of leprosy and improve welfare for the people affected by leprosy. This protocol presents a plan to describe the overall implementation of the Replicability Model and describe the barriers and facilitators encountered in the process. We will also quantify the effect of the program on one of its key aims- early leprosy diagnosis.<br><br>Methods<br><br>We have two Work Packages (WPs). In WP1, we will conduct a process evaluation. This will include three methods i) observations of replicability model implementation teams’ monthly meetings ii) key informant interviews (n=10) and interviews with stakeholders (n=30) iii) observations of key actors (n=15).Our purpose is to describe the implementation process and identify barriers and facilitators to successful implementation.<br><br>WP2 will be a quantitative study to track existing and new cases of leprosy using routinely collected data. If the intervention is successful, we expect to see an increase in cases (with a higher proportion detected at an early clinical stage) followed by a decrease in total cases.<br><br>Conclusion<br><br>This study will enable us to improve and disseminate the Replicability Model by identifying factors that promote success. It will also identify its effectiveness in fulfilling one of its aims: reducing the incidence of leprosy by finding and tracking cases at an earlier stage in the disease.
Order of Authors:	Joydeepa Darlong, MBBS MD
	Karthikeyan Govindasamy
	Onaedo Ilozumba
	Sopna Choudhury
	Anjali Shrivastva

Powered by Editorial Manager® and ProduXion Manager® from Aries Systems Corporation

	Frances Griffiths
	Samuel Watson
	Jo Sartori
	Richard Lilford
**Additional Information:**	
**Question**	**Response**
**Financial Disclosure**<br><br>Enter a financial disclosure statement that describes the sources of funding for the work included in this submission. Review the submission guidelines for detailed requirements. View published research articles from *PLOS ONE* for specific examples.<br><br>This statement is required for submission and **will appear in the published article** if the submission is accepted. Please make sure it is accurate.<br><br>**Unfunded studies**<br>Enter: *The author(s) received no specific funding for this work.*<br><br>**Funded studies**<br>Enter a statement with the following details:<br>• Initials of the authors who received each award<br>• Grant numbers awarded to each author<br>• The full name of each funder<br>• URL of each funder website<br>• Have or will the sponsors or funders play any role in the study design, data collection (when applicable) and analysis, decision to publish, or preparation of the manuscript?<br>• **NO** - Include this sentence at the end of your statement: *The funders had and will not have a role in study design, data collection and analysis, decision to publish, or preparation of the manuscript.*<br>• **YES** - Specify the role(s) played.<br><br>* typeset	This research was funded by the National Institute for Health Research (NIHR: 200132) using UK aid from the UK Government to support global health research. The views expressed in this publication are those of the author(s) and not necessarily those of the NIHR.
**Competing Interests**<br><br>Use the instructions below to enter a competing interest statement for this submission. On behalf of all authors, disclose any competing interests that	I have read the journal's policy and three authors (Joydeepa Darlong, Karthikeyan Govindasamy and Anjali Shrivastva),of this manuscript have the following competing interests: They work for the implementing organization TLMTI

could be perceived to bias this work—acknowledging all financial support and any other relevant financial or non-financial competing interests.<br><br>This statement is required for submission and **will appear in the published article** if the submission is accepted. Please make sure it is accurate and that any funding sources listed in your Funding Information later in the submission form are also declared in your Financial Disclosure statement.<br><br>View published research articles from *PLOS ONE* for specific examples.<br><br>**NO authors have competing interests**<br><br>Enter: *The authors have declared that no competing interests exist.*<br><br>**Authors with competing interests**<br><br>Enter competing interest details beginning with this statement:<br><br>*I have read the journal's policy and the authors of this manuscript have the following competing interests: [insert competing interests here]*<br><br>* typeset	
**Ethics Statement**<br><br>Enter an ethics statement for this submission. This statement is required if the study involved:<br><br>• Human participants<br>• Human specimens or tissue<br>• Vertebrate animals or cephalopods<br>• Vertebrate embryos or tissues<br>• Field research<br><br>Write "N/A" if the submission does not require an ethics statement.	The Leprosy Mission Trust India Ethics Committee reviewed and approved the project (C-046/ TLMTI EC/21) entitled: “Evaluation of ‘Replicability’ model project for early detection and treatment of leprosy and estimating the prevalence and severity of disability in the community in Champa district, Chhattisgarh, India on 07/04/2021

Powered by Editorial Manager® and ProduXion Manager® from Aries Systems Corporation

General guidance is provided below. Consult the submission guidelines for detailed instructions. **Make sure that all information entered here is included in the Methods section of the manuscript.**<br><br>**Format for specific study types**<br><br>**Human Subject Research (involving human participants and/or tissue)**<br>• Give the name of the institutional review board or ethics committee that approved the study<br>• Include the approval number and/or a statement indicating approval of this research<br>• Indicate the form of consent obtained (written/oral) or the reason that consent was not obtained (e.g. the data were analyzed anonymously)<br><br>**Animal Research (involving vertebrate animals, embryos or tissues)**<br>• Provide the name of the Institutional Animal Care and Use Committee (IACUC) or other relevant ethics board that reviewed the study protocol, and indicate whether they approved this research or granted a formal waiver of ethical approval<br>• Include an approval number if one was obtained<br>• If the study involved *non-human primates*, add *additional details* about animal welfare and steps taken to ameliorate suffering<br>• If anesthesia, euthanasia, or any kind of animal sacrifice is part of the study, include briefly which substances and/or methods were applied<br><br>**Field Research**<br><br>Include the following details if this study involves the collection of plant, animal, or other materials from a natural setting:<br>• Field permit number<br>• Name of the institution or relevant body that granted permission	
**Data Availability**<br><br>Authors are required to make the data	N/A - the protocol does not report results

Powered by Editorial Manager® and ProduXion Manager® from Aries Systems Corporation

underlying their research findings fully available, without restriction.<br><br>If the manuscript reports pilot or preliminary results, the data underlying those results must be made available at the time of publication. A Data Availability Statement describing where the data can be found is required at submission. Your answers to this question constitute the Data Availability Statement and will be published in the article, if accepted. When legal or ethical requirements prohibit public data sharing, authors must indicate how researchers may obtain access to the data. See the PLOS Data Policy and FAQ for detailed information.<br><br>**Important:** Stating 'data available on request from the author' is not sufficient. If your data are only available upon request, select 'No' for the first question and explain your situation in the text box.<br><br>For protocols without pilot or preliminary data, authors are strongly encouraged to state how they plan to share research data from their study when it is completed or published.<br><br>Do the authors confirm that EITHER the protocol does not report results OR all data underlying the preliminary results will be made fully available without restriction?	
**Describe where the data may be found in full sentences. If you are copying our sample text, replace any instances of XXX with the appropriate details.**<br><br>• If **no pilot data are reported** in the manuscript, please enter the following: *No datasets were generated or analysed during the current study. All relevant data from this study will be*	No datasets were generated or analyzed during the current study. All relevant data from this study will be made available upon study completion.

made available upon study completion.<br>• If the manuscript reports pilot data **held in a public repository**, include URLs, accession numbers or DOIs. If this information will only be available after acceptance, indicate this by ticking the box below. For example: *All XXX files are available from the XXX database (accession number(s) XXX, XXX.).*<br>• If the manuscript reports pilot data contained **within the manuscript and/or Supporting Information files**, enter the following:<br>*All relevant data are within the manuscript and its Supporting Information files.*<br>• If neither of these applies but you are able to provide **details of access elsewhere**, with or without limitations, please do so. For example:<br><br>*Data cannot be shared publicly because of [XXX]. Data are available from the XXX Institutional Data Access / Ethics Committee (contact via XXX) for researchers who meet the criteria for access to confidential data.*<br><br>*The data underlying the results presented in the study are available from (include the name of the third party and contact information or URL).*<br>• This text is appropriate if the data are owned by a third party and authors do not have permission to share the data.<br><br>For study protocols with data management or sharing plans, authors are encouraged to briefly describe in their Data Availability Statement how data they generate will be made accessible when the study is completed. For example:<br>• Deidentified research data will be made publicly available when the study is completed and published.<br><br>* typeset	
Additional data availability information:	Tick here if the URLs/accession numbers/DOIs will be available only after acceptance of the manuscript for publication so that we can ensure their inclusion before

Powered by Editorial Manager® and ProduXion Manager® from Aries Systems Corporation

	publication.

Manuscript
Click here to access/download;Manuscript;Protocol paper Final (1).docx

An Evaluation Protocol of 'Replicability Model' project for detection and treatment of leprosy and related disability in Chhattisgarh, India.

Joydeepa Darlong[1], Karthikeyan Govindasamy[1], Onaedo Ilozumba[2], Sopna Choudhury[2], Anjali Shrivastva[1], Frances Griffiths[3], Samuel Watson[2], Jo Sartori[2], Richard Lilford[2]

[1] The Leprosy Mission Trust India, 16 Pandit pant Marg , CNI Bhavan , New Delhi, 110001

joydeepa.darlong@leprosymission.in, karthikeyan.g@leprosymission.in, anjali.shrivastva@leprosymission.in.

[2]Institute of Applied Health Research, College of Medical and Dental Sciences, University of Birmingham, Edgbaston, Birmingham, B15 2TT, UK.

r.j.lilford@bham.ac.uk; s.m.choudhury.1@bham.ac.uk; j.m.Sartori@bham.ac.uk; s.i.watson@bham.ac.uk; u.ilozumba@bham.ac.uk

[3]Warwick Medical School, A-155, University of Warwick, UK f.e.griffiths@warwick.ac.uk

Abstract

Introduction

People affected by leprosy are at increased risk of impairments and deformities from peripheral nerve damage. This mostly occurs if diagnosis and treatment is delayed and contributes to continued transmission within the community. Champa district of Chhattisgarh state in India is an endemic area with the highest national annual case detection and disability rates for leprosy. The Replicability Model is a system strengthening intervention implemented by the Leprosy Mission Trust India in Champa that aims to promote early diagnosis and treatment of leprosy, improve on-going management of the effects of leprosy and improve welfare for the people affected by leprosy. This protocol presents a plan to describe the overall implementation of the Replicability Model and describe the barriers and facilitators encountered in the process. We will also quantify the effect of the program on one of its key aims- early leprosy diagnosis.

Methods

We have two Work Packages (WPs). In WP1, we will conduct a process evaluation. This will include three methods i) observations of replicability model implementation teams' monthly meetings ii) key informant interviews (n=10) and interviews with stakeholders (n=30) iii) observations of key actors (n=15).Our purpose is to describe the implementation process and identify barriers and facilitators to successful implementation.

WP2 will be a quantitative study to track existing and new cases of leprosy using routinely collected data. If the intervention is successful, we expect to see an increase in cases (with a higher proportion detected at an early clinical stage) followed by a decrease in total cases.

Conclusion

This study will enable us to improve and disseminate the Replicability Model by identifying factors that promote success. It will also identify its effectiveness in fulfilling one of its aims: reducing the incidence of leprosy by finding and tracking cases at an earlier stage in the disease.

Keywords:

System strengthening, Early detection, Leprosy, Disability, Low and middle-income countries

BACKGROUND

Leprosy is a neglected tropical disease, caused by a slow growing bacterium called the Mycobacterium leprae [1]. The condition is characterized by anaesthetic skin lesions and damage to peripheral nerves. The clinical presentation varies across a wide spectrum from tuberculoid to lepromatous leprosy. Treatment of leprosy constitutes of a combination of antibiotics (Rifampicin, Dapsone and Clofazimine) called multi drug therapy [2]. If not treated timely and appropriately, it can cause irreversible damage to the peripheral nerves leading to loss of sensation and muscle function, resulting in visible deformities, functional impairments, and disabilities with serious health, social and economic consequences[3].

The number of active cases (quantum of infection) is the major determinant of leprosy transmission in the community. Because of the long incubation period of Mycobacterium leprae, delayed detection results in prolonged contact of infected patients with the other members of thus increasing transmission [4]. Delays in diagnosis and treatment of leprosy occur at three levels: individual, community and health facility. At the individual and community level, a lack of awareness about leprosy and prevailing stigma surrounding the disease results in concealment and delay in reporting to health centres [5]. At the health facility level, lack of clinical expertise and limited resources lead to further delay and subsequent complications, such as inflammatory reactions and peripheral nerve damage [6].

Since the introduction of multi drug therapy in the early eighties, there has been marked reduction in the number of cases of leprosy. The World Health Assembly Resolution in 1991 to “eliminate leprosy as a public health problem by 2000” was followed by a 90% decrease in the prevalence of leprosy, which dropped to less than 1 in 10,000 at the global level [7]. By the end of 2005 most countries had eliminated leprosy at the national level. However, India continues to face challenges with leprosy elimination. In 2020, India registered 114,451 cases. The prevalence rate of 0.4 per 10,000 population represents 57% of the global burden [8]. Of

the new cases detected, 58.1% were multibacillary, 5.8% were in children less than 14 years of age, and 2.4% of all cases had visible deformities. There are more than 3 million people with leprosy deformities that need attention and care [9]. A high national case detection rate implies continued transmission of the disease, and a high grade 2 deformity rate is evidence of delayed detection. Thus, finding new leprosy cases early is the accepted cornerstone of leprosy control strategies [10].The National Leprosy Eradication Program in India is responsible for the diagnosis and treatment of the disease and for prevention and management of disability due to leprosy [11]. National program activities and plans are formulated centrally and implemented by state authorities. The National Leprosy Eradication Program has developed multiple initiatives to promote early detection of leprosy. However, they are rarely evidence-based or integrated into national health programs. Interventions that are evidence-based and implemented through the national health program for leprosy are more likely to be sustainable and replicable elsewhere than stand-alone interventions delivered by an outside agency (16).

There is therefore a need for a program that comprehensively addresses early diagnosis of leprosy, prevention of leprosy related disability, and socioeconomic advancement and inclusion of people affected by leprosy. Responding to this need, the Leprosy Mission Trust India has implemented a project in the district of Champa, Chhattisgarh state, named the Replicability Model project which seeks to strengthen the activities of the government in the three pillars of zero leprosy: transmission, disability, and discrimination.

In summary, the Replicability Model has two noteworthy features. First it covers leprosy management as a whole covering prevention of spread, management of complications and improving well-being through social integration. Second, it is not a stand-alone NGO delivered program, but it is delivered through the public health services, a feature that should make it replicable.

This manuscript presents the protocol for an evaluation of the Replicability Model project (2020-2024). The research aims to:

i. Find out how the overall Replicability Model project is implemented and identify barriers and facilitators encountered along the way.
ii. Evaluate the first specific aim of the Replicability Model project by measuring effects on early diagnosis of leprosy.

A team from the University of Birmingham and members from The Leprosy Mission Trust India, who are not involved in the implementation of the Replicability Model project, under the NIHR Research and Innovation for Global Health Transformation (RIGHT) grant, will conduct the evaluation.

Intervention Description – The Replicability Model (RM) project

The RM project is co-developed by The Leprosy Mission Trust India (TLMTI) and the ministry of Health and funded by the Leprosy Mission England & Wales. It is implemented by TLMTI in Janjgir-Champa district, Chhattisgarh. This project has an implementation timeline of 5 years (2020-2024), and project evaluation will occur concurrently.

The main objective of the RM project is to strengthen the health system to reduce disability rate amongst people affected by leprosy in Janjgir-Champa District. The RM project has three aims:

1) improve early detection of leprosy
2) improve the prevention and management of leprosy and reduce leprosy disabilities
3) improve access to rights and entitlements and reduce stigma and discrimination to promote inclusion

Our hypothesis is that, due to its comprehensive nature and integration into the existing governance systems, the RM project will prove to be effective, sustainable, and transferable.

The overall framework of the intervention is illustrated in (Figure 1) and described in detail according to the template for reporting interventions (TiDiER) document in Supplementary File 1. The project involves several mission critical activities, including health promotion, health worker education, data gathering, health care systems (surveillance, contact tracing, referral) and provision of equipment. The delivery of the RM project involves health workers namely Mitanins (community health workers), male volunteers, Child Health Screening and Early Intervention Services (Rashtriya Bal Swasthya Karyakram) staff and medical officers.

Figure 1: Overall framework of interventions of Replicability Model in Janjgir-Champa district.

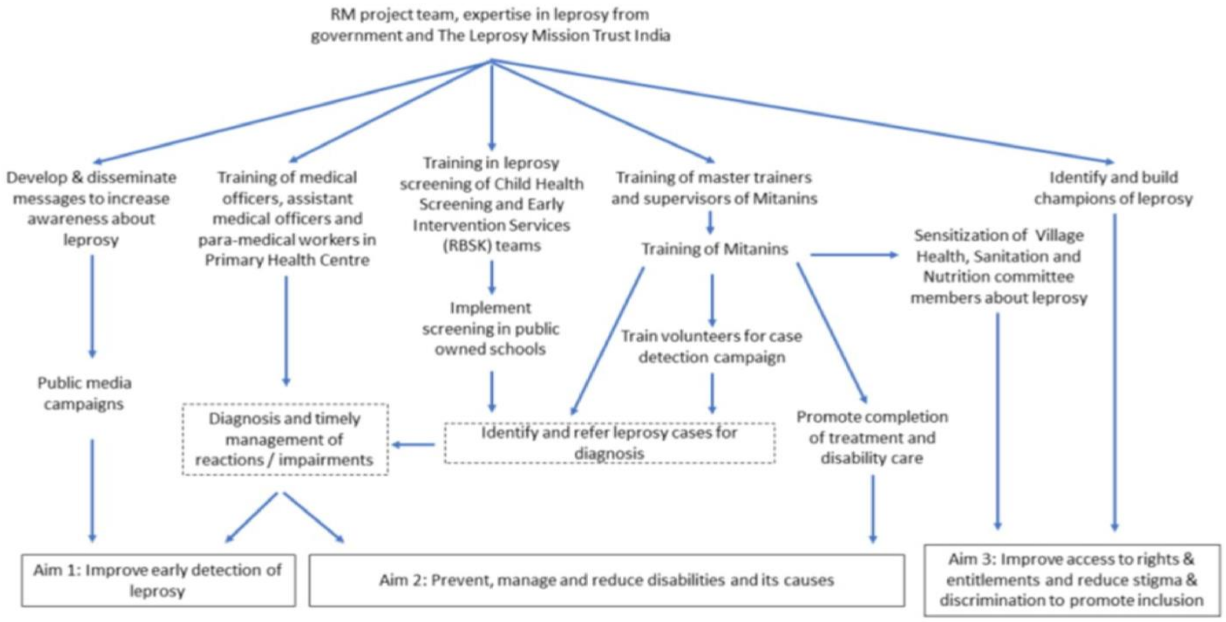

Training sessions will be ‘cascaded’ to front line staff through a train-the-trainers program as follows: 180 Masters trainers from the study site will attend a 1-day trainers’ program . These master trainers will then be responsible for training Mitanins, male volunteers and Rashtriya Bal Swasthya Karyakram team members as follows:

i) Mitanins: Mitanins (n=3,791) will attend training sessions delivered by the master trainers. The sessions will be delivered to groups of approximately 20 Mitanins and address leprosy detection and the referral process to health facilities for diagnosis and treatment.

ii) Team members and male volunteers: 10 members of the Rashtriya Bal Swasthya Karyakram teams and male volunteers will be trained in a one-off training program in the district headquarters. If needed, refresher courses will be planned.

iii) Medical officers: The medical officers (n=7) and assistant medical officers (n=65) posted in the Primary Health Centres will receive training on diagnosis, confirmation, and management of cases for leprosy and its [illegible]nplications.

METHODS

Study Setting

Chhattisgarh state (Fig 2) has 27 districts with a population of 28 million as per census 2011 [12]. With a literacy rate of 70% it is one of the six worst performing states in India. About 45% of the state’s population lives below the poverty line, with a major portion (31%) belonging to Scheduled Caste/Scheduled Tribe category[13]. Tribal populations in India are often disadvantaged and report poor health outcomes, limited access to infrastructure and financial opportunities[14].

Figure 2: Map of Chhattisgarh State and District of Janjgir-Champa

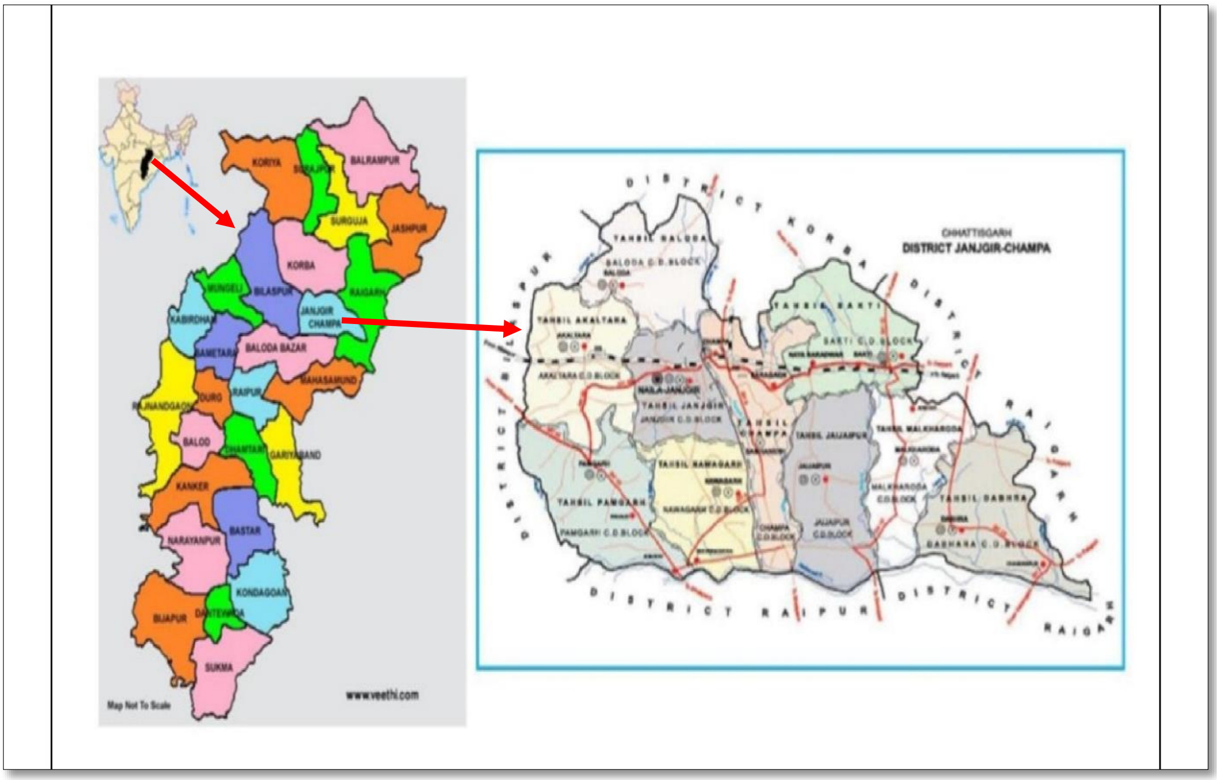

We will conduct our study in the district of Janjgir-Champa in Chhattisgarh state. The state bears the highest burden of leprosy cases and leprosy related Grade 2 Disability. In 2018-2019, a total of 724 new leprosy cases were identified in the district, representing 9% of the total state burden of new leprosy cases. The Annual New Case Detection Rate of 43.69 per 100,000 population for the year April 2016 to March 2017 for leprosy is about three times the national average [15].

The research study will be carried out in two-work packages:

Work Package 1 (WP1): Process Evaluation of the RM project, observing factors that impede and promote its implementation.

Work Package 2 (WP2): Quantitative Evaluation of effectiveness in detecting new cases.

We will conduct a further nested study in which we will study ulcer prevalence and intervene with the intention of reducing the burden of ulcers in the community. This third study will be nested in the Replicability model and will be described in a separate protocol.

WP1 – Process Evaluation of the RM project

WP1 will evaluate the overall RM project as implemented in Janjgir-Champa, Chhattisgarh. The RM project is complex[16] and, as stated, will be implemented within the existing health system and the communities it serves . The aim is to study how the intervention is implemented and identify barriers and enablers to implementation[17]

Identification and Evaluation of Mission Critical Activities of the RM Project.

We define mission critical activities as those that are essential to achieve the project aims. To identify and refine mission critical activities, we will conduct key informant interviews with individuals from stakeholder groups including TLMTI staff, Mitanins, Rashtriya Bal Swasthya Karyakram team members, medical officers, primary healthcare workers and community representatives (n=10).

The mission critical activities will be evaluated in three ways: observation of team meetings, interviews with key stakeholders and observation of key actors in their workplaces.

1. *Observation of RM Implementation Team Meetings:*The research team will observe monthly meeting where senior staff discuss the implementation of the RM project and make decisions. The researcher will take anonymised field notes of monthly meetings.
2. *Interviews with Key Stakeholders:* The interview schedule will be developed and piloted with representatives from the relevant stakeholder groups (n=10). Semi-structured interviews will be conducted with 8-10 participants in each of the three groups responsible for intervention implementation as described above and persons

affected by leprosy (n=30). Interview guides will cover the following topics: improvement in knowledge and ability to screen, diagnose, refer, and manage cases thus achieving early detection of leprosy.

3. *Observations of Key Actors*: We will observe 15 Mitanin daily sessions and 5 Rashtriya Bal Swasthya Karyakram team members (n= 20), in their workplace using checklists to evaluate the fidelity of the mission critical activities. The researcher will be trained to observe and record key events including the interactions between health workers and community members, enactment of the mission critical activities.

Data Analysis

Data analysis of three data types will focus on how the mission critical activities are (or are not) undertaken by the personnel within their context and factors that facilitative or impede implementation We will examine how different health service or community context affect the process. We will use the theoretical approach summarised by May[18][19].

The key analysis questions will be iteratively refined during process evaluation data collection. The basic framework will include questions such as:

- How, where, when and with whom do the actors enact the mission critical activities, how does this vary and why?
- What do they negotiate in advance to enable them to enact the mission critical activities (e.g., changes in working patterns or systems; changing attitudes in communities), how easy is this negotiation and what prevents change?
- How do the mission critical activities become normalised within their daily activities/context?

- How do relationships within their context change to enable mission critical activities and their sustainability?

WP2 – Evaluation of RM project aimed at early detection of new cases (aim 1 of RM project)

In this WP a time series analysis will be conducted in the intervention district with a contemporaneous time series in a control district of Raigarh with similar epidemiological trends as the study district. The trends in epidemiological indicators for the last 6 years will form the baseline situation of the disease in the intervention and control areas. Data on all patients registered for treatment is available from the community health centres and recognized non-government organization (NGO) owned hospitals (Fig 3).

Fig 3 : shows the flow of data from Primary Health Centres to State level

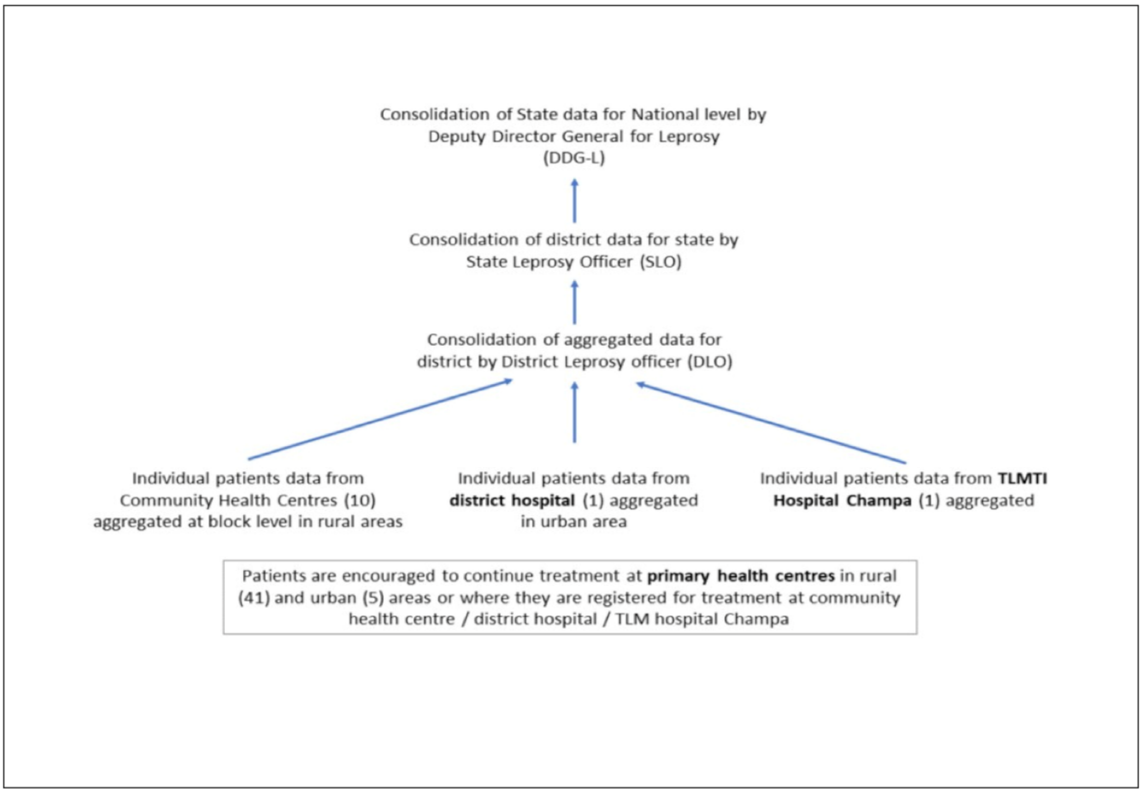

Data

The pre-intervention period will be 2015 – 2020. The data for the post intervention period will be 2021 and 2024. Individual patient data is collected by the NLEP staff of Community Health Centres routinely using standard individual patient forms (see Appendix 1) and treatment registers (Appendix 2).

The key epidemiological indicators for data analysis are shown in Table 1 Timeline for data collection activities is presented in Figure 4

Table 1 : Key Epidemiological Indicators

Indicators	Numerator	Denominator	Source of data
Prevalence rate (PR) per 10,000 population	Number of cases under treatment at the end of the reporting period	Total population of the area where the data is drawn	Treatment register
Annual new case detection rate (ANCDR) per 100,000 population	Number of new leprosy cases detected during the reporting period		Treatment register
Proportion of patients with grade II disability among new cases	Number with grade II disability at diagnosis among new cases detected	Number of new leprosy cases detected during the reporting period	Individual patient card*
Proportion of child cases (<15 years) among new cases	Number of child cases (<15 years) among new cases detected		
Proportion of MB cases disability among new cases	Number of MB cases among new cases detected		
Proportion of female cases among new cases	Number of female cases among new cases detected		
Proportion of SC/ST cases among new cases	Number of SC/ST cases among new cases detected		

Figure 4: **Timeline for evaluation of early case detection**

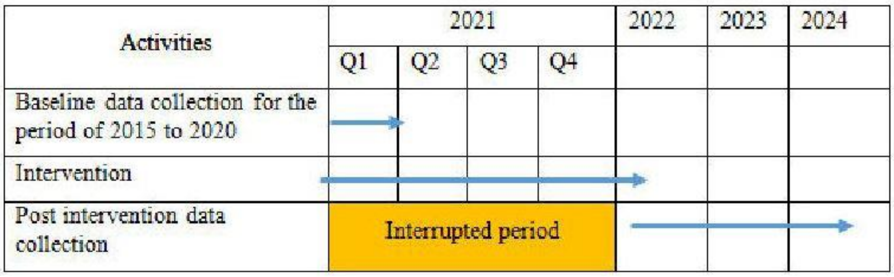

Data Entry

This data will be extracted, and anonymous data entered into the project database on a monthly basis by the NIHR RIGHT research project staff. Range limits and logic checks (e.g., for conflicting responses) will be built into the electronic data collection forms to prevent

erroneous data entry. Initial data from the first ten participants will be cross-checked by the local lead investigator to ensure that full and accurate data are collected.

Data Analysis

The objectives of the analysis are to assess the impact over time of the intervention on:

(i) the incidence rate of (diagnosed) leprosy and

(ii) the incidence rate of leprosy by grade of disability.

The incidence rate of (diagnosed leprosy): The analysis will be at the block-month level. For block $i = 1, \dots, N$ at time $t = 1, \dots, T$ the number of new cases is $y_{jt} \in \mathbb{N}$, which we model as Poisson distributed with intensity λ_{jt}:

$$y_{jt} \sim Poisson(\lambda_{jt})$$

$$\lambda_{jt} = e_{jt} \exp\left(\beta_0 + \beta_1' x_{jt} + \gamma_t I(d_{jt} = 1) + \alpha_j + \tau_t\right) \quad (1)$$

where x_{jt} is a vector of block level covariates including demographic indicators like the age distribution of the population, d_{jt} is an indicator for whether block j has started the implementation of the intervention at time t, $\alpha_j \sim N(0, \sigma_\alpha^2)$ is a block-level random effect, and τ_t is a set of monthly indicators. We include an offset term e_{jt}, which is the size of the population in the block so that the exponential term represents a relative risk. We allow the treatment effect γ_t to vary by time as we expect the incidence to first increase and then decrease post implementation of the intervention. The roll-out of the intervention will take a number of months so there will be a number of "cross-over" months. We will make comparisons of the parameter of interest over the whole period and also compare strictly pre- and post-intervention estimates of the parameters as well as intervention time interactions.

The incidence by grade of disability, we use the same model as specified in Equation but set the outcome to be the number of cases at each level of disability. Post intervention we

hypothesise that the incidence of cases with severe disability will decrease and those without severe disability to increase in the first instance. Over-time, cases will decrease if the intervention is successful.

DISCUSSION

The RM Project is an ambitious program targeting leprosy. It is unusual, arguably unique, in its philosophy and scope. First, it is not a stand-alone program. Rather it is embedded in and owned by the health service although largely funded by an NGO. Second, it goes beyond health services, involving schools and aiming to improve case detection among school children and third, it has multiple aims addressing the three zeros of transmission, disability, and discrimination of leprosy.

The RM project as described in this protocol has the potential to influence the incidence rate of leprosy. The first indication of an effective intervention would be an increase in the total number of cases or new cases detected/reported, downstaging of the disease and subsequently a decrease in total number of leprosy cases.

This study both capitalises on and contributes to the planned roll out of the RM project. A key strength of this study is its mixed methods approach which will contribute to an understanding not only of outcomes but also processes. Our results will be useful in informing leprosy programs elsewhere and we hope our methods will be useful to inform evaluation of other multi-purpose, multi-component, across agency interventions. The evaluation will provide additional evidence relating to the effectiveness of the case-finding approach of RM project by conducting a comprehensive search for people at risk of leprosy and their accurate diagnosis by the physician in the health centres.

However, there are also study limitations. The evaluation team works for the same organization as the implementation team. While research and management processes will be implemented

to ensure that the evaluation is independent, this cannot be completely guaranteed. However, the evaluation team will document all deviations from the protocol, and these will be included in future reporting. In addition, in WP3 (a nested study to be described separately), we will estimate the prevalence of disabilities among people affected by leprosy in the study area. This will provide baseline data to effectively plan, develop, and evaluate a follow-on study of an intervention to reduce the prevalence of disabilities in eyes, hands, and feet through enhanced self-care in line with aim 2 of the RM project.

ETHICAL APPROVAL AND CONSENT TO PARTICIPATE

The research will be performed in accordance with the Declaration of Helsinki for Human Research of the World Medical Association and approval has been granted by the University of Birmingham Biomedical and Scientific Research Ethics Committee (BSREC) and locally in India by The Leprosy Mission Trust India Ethics committee.

Eligible people will be provided with a Participant Information Sheet in local languages. Information will be provided verbally for participants who are non-literate. Written informed consent will be obtained from all participants; or thumb/fingerprints will be requested in lieu of a signature if necessary. Translated consent forms will be back-translated according to the WHO recommendations (11) for quality assurance purposes. Participants will be free to withdraw at any time.

.

COMPETING INTERESTS

The authors have no competing interests.

FUNDING

This research was funded by the National Institute for Health Research (NIHR: 200132) using UK aid from the UK Government to support global health research. The views expressed in this publication are those of the author(s) and not necessarily those of the NIHR.

Authors' contributions

JD, KG, OI, SC, AS, SW FG, and JS contributed to the development and editing of the manuscript. RJL: Director of the NIHR RIGHT funded project at University of Birmingham and contributed to the conception of this evaluation, and critically evaluated the intellectual content.

Acknowledgements

We acknowledge the Replicability Model Implementation team for their cooperation in the development of the evaluation protocol. We would also like to acknowledge Sian Arulanantham from The Leprosy Mission England and Wales for her continued support with all our work.

Additional Files

Appendix 1 – Baseline data collection for early detection of leprosy (WP2)

month and year	district	Block name	Number of New cases	Number of MB cases	Number of child cases	Number of female cases	Number of Grade 1 disability	Number of Grade 2 disability	Number of cases from SC/ST
Apr-15	Champa								
May -15									
Jun – 15									
Jul – 15									
Aug – 15									
Sep – 15									
Oct – 15									
Nov – 15									
Dec - 15									
Jan – 16									
Feb – 16									
Mar - 16									
Mar-20	Champa								

Appendix 2 : Treatment register

TREATMENT REGISTER FOR OTHER CASES

U.L.F. 02/B

PHC ________________________________ Block PHC/CHC _________________________________

Districts ________________________________ State/UTs _________________ Fiscal Year __________

Reg. No.	Sub Centre	Name	Address with mobile tel. number	Age	Sex M/F	ST / SC	PB / MB	Disability Grade I / II	Date of First Dose	Date of Subsequent doses											Date of RFT
										2	3	4	5	6 (PB Final)	7	8	9	10	11	12 (MB Final)	

* - Category of case – Relapse, re-entered for treatment completion, referred and changing in classification of MB / PB

REFERENCES

[1] Rao PN, Rao PN. Leprosy: The challenges ahead for India. Journal of Skin and Sexually Transmitted Diseases 2021;3:106–10. https://doi.org/10.25259/JSSTD_42_2021.

[2] Leprosy: overcoming the remaining challenges n.d. https://apps.who.int/iris/handle/10665/164331 (accessed March 22, 2022).

[3] Health Organization W. WORLD REPORT ON DISABILITY WHO Library Cataloguing-in-Publication Data. 2011.

[4] Directorate General Of Health Services n.d. https://dghs.gov.in/content/1349_3_NationalLeprosyEradicationProgramme.aspx (accessed March 18, 2022).

[5] Supporting leprosy elimination in India n.d. https://www.who.int/india/news/detail/01-02-2022-supporting-leprosy-elimination-in-india (accessed March 18, 2022).

[6] M H, N G, K T. Factors contributing to the delay in diagnosis and continued transmission of leprosy in Brazil—an explorative, quantitative, questionnaire based study. PLoS Negl Trop Dis n.d.;10:e0004542.

[7] Smith WC, van Brakel W, Gillis T, Saunderson P, Richardus JH. The Missing Millions: A Threat to the Elimination of Leprosy. PLoS Neglected Tropical Diseases 2015. https://doi.org/10.1371/journal.pntd.0003658.

[8] Weekly Epidemiological Record (WER), 10 September 2021, Vol. 96, No. 36 (pp. 421–444) [EN/FR] - World | ReliefWeb n.d. https://reliefweb.int/report/world/weekly-epidemiological-record-wer-10-september-2021-vol-96-no-36-pp-421-444-enfr (accessed March 18, 2022).

[9] Rao Pn, Suneetha S. Current situation of leprosy in India and its future implications. Indian Dermatology Online Journal 2018;9:83. https://doi.org/10.4103/idoj.idoj_282_17.

[10] Enhanced global strategy for further reducing the disease burden due to leprosy (2011-2015): plan period: 2011-2015) n.d. https://apps.who.int/iris/handle/10665/205004 (accessed March 22, 2022).

[11] Rao Pn, Suneetha S. Current situation of leprosy in India and its future implications. Indian Dermatology Online Journal 2018;9:83. https://doi.org/10.4103/idoj.idoj_282_17.

[12] Census of India Website : Office of the Registrar General & Census Commissioner, India n.d. https://censusindia.gov.in/2011-Common/Archive.html (accessed January 5, 2022).

[13] Chhattisgarh Poverty, Growth & Inequality n.d.

[14] Interface T, Training V, Manifestations V, Equity G, Culture T, Genius T. Draft The National Tribal Policy n.d.

[15] In 2021-22, Over 2,110 Cases Of Leprosy Reported In Cg | Raipur News - Times of India n.d. https://timesofindia.indiatimes.com/city/raipur/in-2021-22-over-2110-cases-of-leprosy-reported-in-cg/articleshow/89859837.cms (accessed June 29, 2022).

[16] Moore GF, Audrey S, Barker M, Bond L, Bonell C, Hardeman W, et al. Process evaluation of complex interventions: Medical Research Council guidance. BMJ 2015;350. https://doi.org/10.1136/BMJ.H1258.

[17] Damschroder LJ, Aron DC, Keith RE, Kirsh SR, Alexander JA, Lowery JC. Fostering implementation of health services research findings into practice: A consolidated

framework for advancing implementation science. Implementation Science 2009;4:1–15. https://doi.org/10.1186/1748-5908-4-50/TABLES/1.

[18] May CR, Johnson M, Finch T. Implementation, context and complexity. Implementation Science 2016;11:1–12. https://doi.org/10.1186/S13012-016-0506-3/FIGURES/1.

[19] Pope C, Ziebland S, Mays N. Qualitative research in health care: Analysing qualitative data. BMJ : British Medical Journal 2000;320:114. https://doi.org/10.1136/BMJ.320.7227.114.

Figure 1

Click here to access/download;Figure;RM Evaluation protocol paper Fig 1.jpg

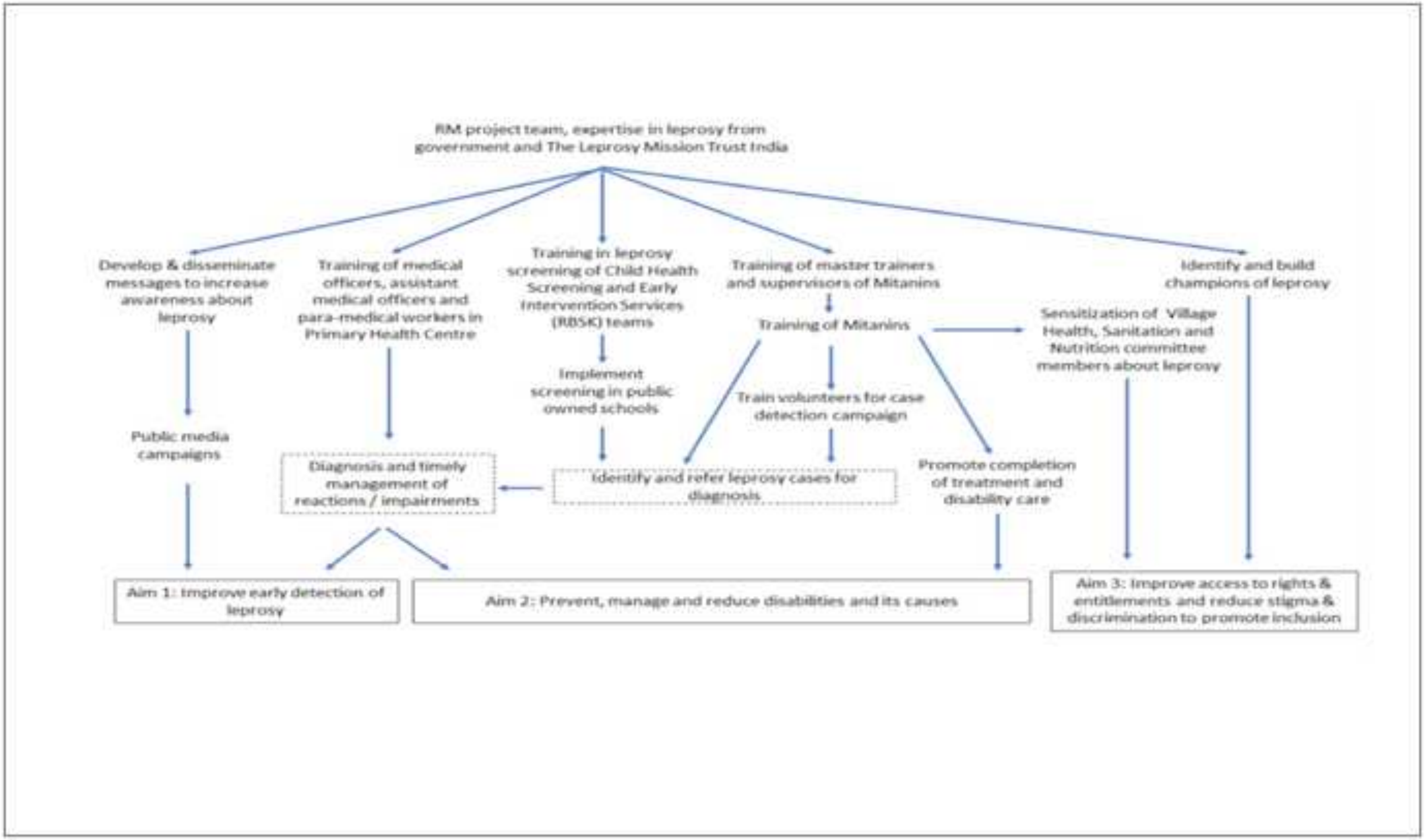

Figure 2

Click here to access/download;Figure;RM Evaluation protocol paper Fig 2.jpg

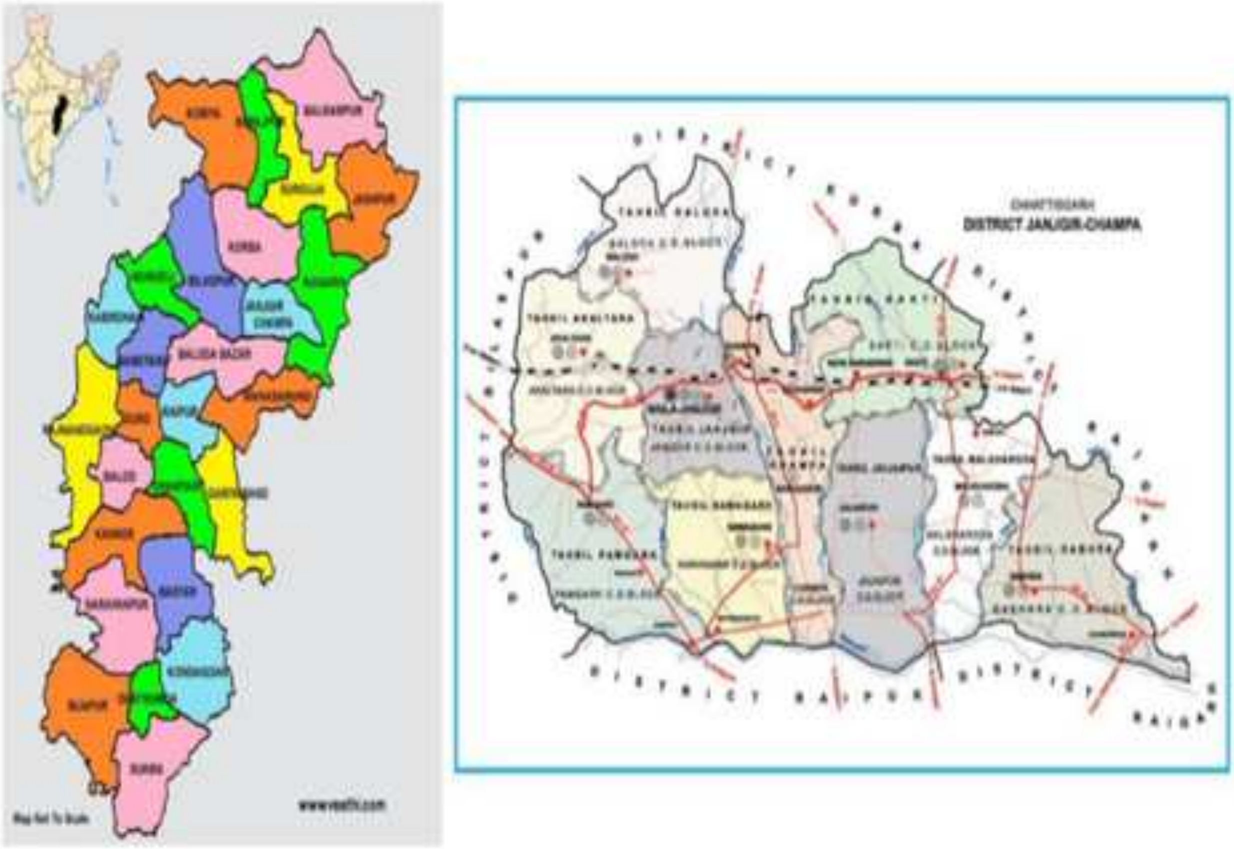

Figure 3

Click here to access/download;Figure;RM Evaluation protocol paper Fig 3.jpg

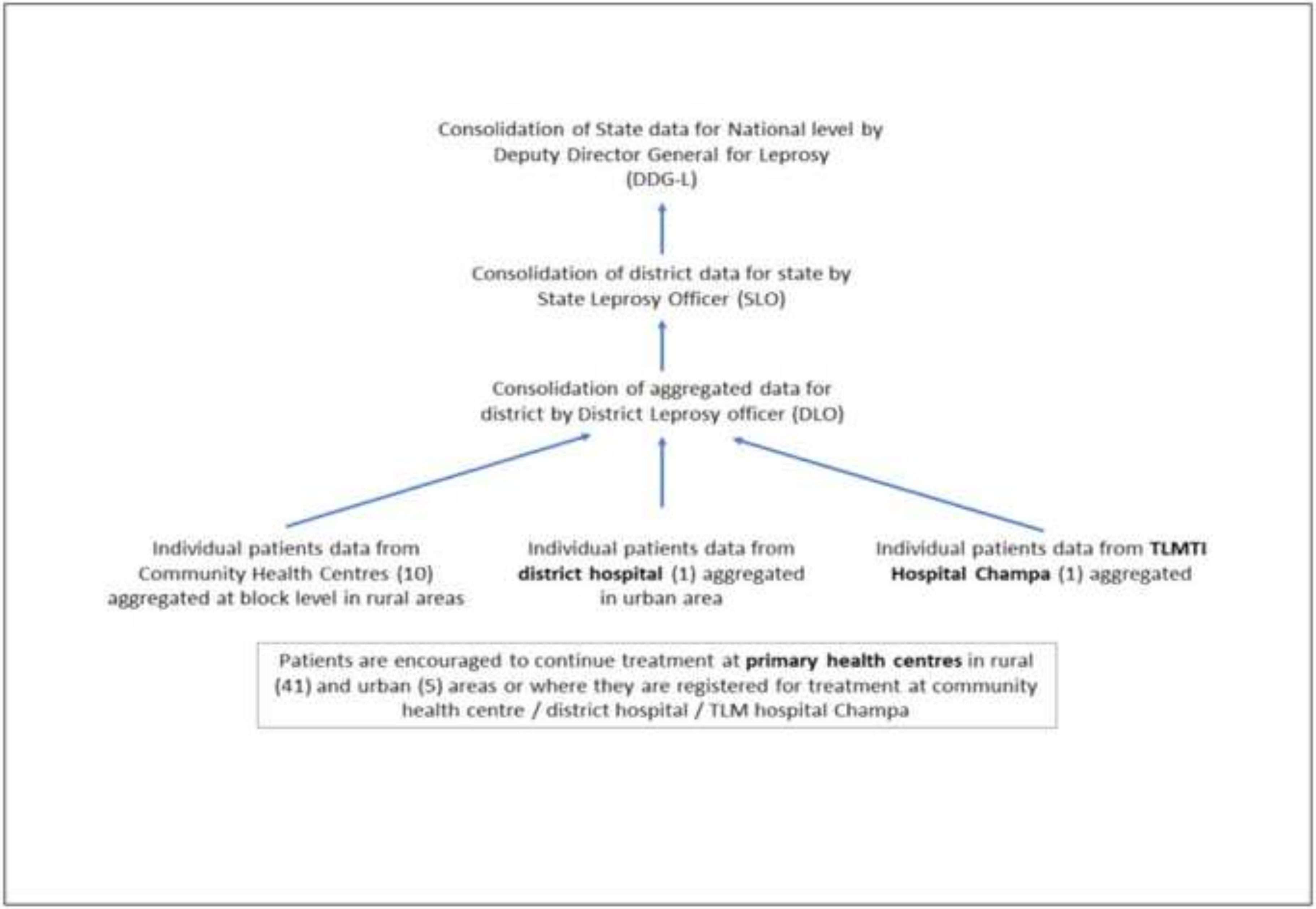

Figure 4 Click here to access/download;Figure;RM Evaluation protocol paper Fig 4.jpg

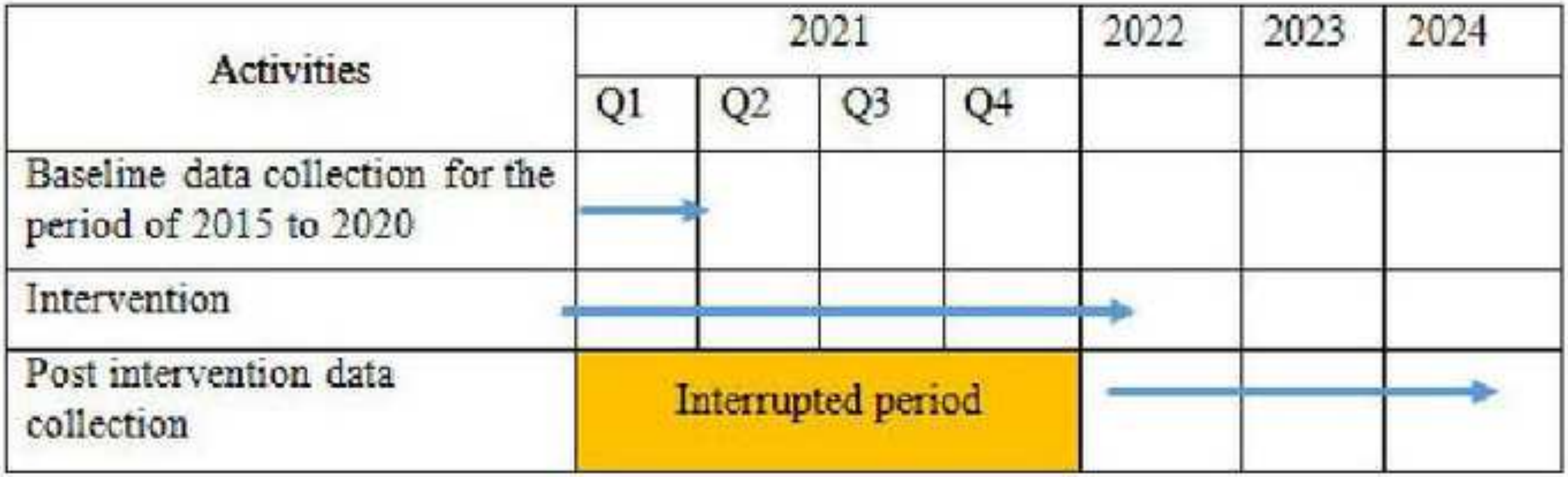

Activities	2021				2022	2023	2024
	Q1	Q2	Q3	Q4			
Baseline data collection for the period of 2015 to 2020							
Intervention							
Post intervention data collection	Interrupted period						

Appendix 1

Click here to access/download
Supporting Information
Appendix 1.docx

Click here to access/download
Supporting Information
Appendix 2.docx

TIDieR description of replicable model project interventions

Click here to access/download
Supporting Information
Supplementary file.docx

Click here to access/download
Supporting Information
Study 2 Ethics approval letter.pdf
